# Reactive Oxygen Species Suppress Cardiac Na_V_1.5 Expression through Foxo1

**DOI:** 10.1371/journal.pone.0032738

**Published:** 2012-02-29

**Authors:** Weike Mao, Tao You, Bo Ye, Xiang Li, Henry H. Dong, Joseph A. Hill, Faqian Li, Haodong Xu

**Affiliations:** 1 Department of Pathology and Laboratory Medicine, University of Rochester Medical Center, Rochester, New York, United States of America; 2 Aab Cardiovascular Research Institute, University of Rochester Medical Center, Rochester, New York, United States of America; 3 Division of Cardiology, Second Affiliated Hospital of Soochow University, Suzhou, Jiangsu, People's Republic of China; 4 Division of Immunogenetics, Rangoes Research Center, Children's Hospital of Pittsburgh of UPMC, Pittsburgh, Pennsylvania, United States of America; 5 Division of Cardiology, University of Texas Southwestern Medical Center, Dallas, Texas, United States of America; Ospedale Pediatrico Bambino Gesu', Italy

## Abstract

Na_V_1.5 is a cardiac voltage-gated Na^+^ channel αsubunit and is encoded by the *SCN5a* gene. The activity of this channel determines cardiac depolarization and electrical conduction. Channel defects, including mutations and decrease of channel protein levels, have been linked to the development of cardiac arrhythmias. The molecular mechanisms underlying the regulation of Na_V_1.5 expression are largely unknown. Forkhead box O (Foxo) proteins are transcriptional factors that bind the consensus DNA sequences in their target gene promoters and regulate the expression of these genes. Comparative analysis revealed conserved DNA sequences, 5′-CAAAACA-3′ (insulin responsive element, IRE), in rat, mouse and human *SCN5a* promoters with the latter two containing two overlapping Foxo protein binding IREs, 5′-CAAAACAAAACA-3′. This finding led us to hypothesize that Foxo1 regulates Na_V_1.5 expression by directly binding the *SCN5a* promoter and affecting its transcriptional activity. In the present study, we determined whether Foxo1 regulates Na_V_1.5 expression at the transcriptional level and also defined the role of Foxo1 in hydrogen peroxide (H_2_O_2_)-mediated Na_V_1.5 suppression in HL-1 cardiomyocytes using chromatin immunoprecipitation (ChIP), constitutively nuclear Foxo1 expression, and RNAi Foxo1 knockdown as well as whole cell voltage-clamp recordings. ChIP with anti-Foxo1 antibody and follow-up semi-quantitative PCR with primers flanking Foxo1 binding sites in the proximal *SCN5a* promoter region clearly demonstrated enrichment of DNA, confirming Foxo1 recruitment to this consensus sequence. Foxo1 mutant (T24A/S319A-GFP, Foxo1-AA-GFP) was retained in nuclei, leading to a decrease of Na_V_1.5 expression and Na^+^ current, while silencing of Foxo1 expression by RNAi resulted in the augmentation of Na_V_1.5 expression. H_2_O_2_ significantly reduced Na_V_1.5 expression by promoting Foxo1 nuclear localization and this reduction was prevented by RNAi silencing Foxo1 expression. These studies indicate that Foxo1 negatively regulates Na_V_1.5 expression in cardiomyocytes and reactive oxygen species suppress Na_V_1.5 expression through Foxo1.

## Introduction

Alterations of ion channel activity play an important role in the development of cardiac arrhythmias. Na_V_1.5 encoded by the *SCN5a* gene is α subunit of the voltage-gated cardiac Na^+^ channel, and this channel activity determines cardiac excitability and conduction. Reactive oxygen species (ROS) are generated during myocardial infarction [1]–[4] and contribute to the development of life-threatening ventricular arrhythmias [5], [6]. It has been shown that alteration of cardiac Na^+^ channel activity immediately following ROS challenge requires activation of Ca^2+^-dependent calmodulin II kinase (CaMKII) [7]. CaMKII regulates Na^+^ channel gating by phosphorylating Na_V_1.5 to increase late Na^+^ current, leading to the prolongation of the action potential duration and development of early afterdepolarization [8]. H_2_O_2_-chronically mediated downregulation of Na^+^ channel activity requires activation of NF-KappaB to decrease Na_V_1.5 expression by inhibiting *SCN5a* promoter activity [9]. Mutation of the NF-KappaB binding site in the *SCN5a* promoter eliminates 42.3% decrease of this promoter activity by H_2_O_2_ in H9C2 myoblasts [9]. These findings indirectly support the notion that other signaling pathways are also significantly involved in the regulation of Na_V_1.5 expression.

Foxo transcription factors form a subclass of the large family of Forkhead proteins characterized by the presence of a “winged-helix” DNA-binding domain called Forkhead box O, which gave the name Foxo proteins to the family [10]. Post-translational modifications such as phosphorylation, acetylation and methylation affect Foxo subcellular localization and transcriptional activity [11]–[13]. Foxo proteins harbor conserved threonine and serines that can be phosphorylated by different kinases, leading to their nuclear retention or exclusion [11]. Akt/protein kinase B (PKB) [14], [15], serum and glucocorticoid-induced kinase 1 (SGK1) [16], cyclin-dependent kinase 2 (CDK2) [17] and IkB kinase (IKK) [18] phosphorylate Foxo proteins, resulting in their nuclear exclusion while c-Jun-N-terminal kinase (JNK) [19] and mammalian sterile 20-like kinase 1 (MST1) [20] phosphorylate Foxo proteins, causing their nuclear retention. Foxo proteins also contain acetylated lysines that can be deacetylated by a NAD-dependent histone deacetylase, silent mating type information regulation 2 homolog (Sirt1), leading to their nuclear retention [12]. Protein arginine methyltransferase 1 (PRMT1) methylates Foxo1 primarily at Arg248 and Arg250 within the consensus Akt phosphorylation motif. These methylations directly abrogate Akt-induced phosphorylation of Foxo1 at Ser253, which in turn results in increased Foxo1 nuclear localization [13]. Accumulating evidence suggests that Foxo1, 3 and 4 are expressed in the heart and they are critical in maintaining cardiac function and mediating cardiac stresses. In the heart, the Foxo family is believed to be involved in diverse activities, including response to oxidative stress [21]–[23], regulation of metabolism [24], modulation of KATP channels [25], promotion of autophagy [26], cell cycle control [27], [28], and commitment to apoptosis [29]. In the nucleus, Foxo proteins target a conserved DNA binding sequence, 5′-GTAAA(C/T)A-3′ [30] or insulin response element (IRE), 5′-CAAAA(C/T)A-3′ [30]–[32] in the promoters of their target genes to affect these gene expression. The end result of Foxo transcriptional activity is generally associated with counteracting oxidative stress and promoting cell cycle arrest and apoptosis [11]. Silico promoter analysis on a list of 354 of the most differentially expressed genes in the thymocyte lineage predicted that the promoter of *SCN5a* has Foxo binding elements [33].

In this study, we tested the hypothesis that H_2_O_2_ suppressing Na_V_1.5 requires the Foxo1 signaling pathway. Our results showed that Foxo1 negatively regulated Na_V_1.5 expression by directly binding the *SCN5a* promoter, and H_2_O_2_-mediated inhibition of Na_V_1.5 expression required Foxo1.

## Materials and Methods

### Ethics

This study was carried out in strict accordance with the recommendations in the Guide for the Care and Use of Laboratory Animals of the National Institutes of Health. The protocol was approved by the University Committee on Animal Resources (UCAR) of University of Rochester Medical Center (UCAR # 2007-060).

### Cell Culture

HeLa cells (obtained from American Type Culture Collection) were maintained in 10 cm plates containing Dulbecco's modified Eagle's medium (high glucose) and 10% fetal bovine serum with 100 U/ml penicillin and 100 µg/ml streptomycin. A cardiomyocyte cell line, designated HL-1, from the AT-1 mouse atrial cardiomyocyte tumor lineage, was kindly provided by Dr. William C. Claycomb at Louisiana State University Health Sciences Center, New Orleans, USA. These cells were cultured as described previously [34]. The cells were cultured in Claycomb medium (JRH Biosciences, KS) supplemented with 10% fetal bovine serum (JRH Biosciences, KS), 2 mM L-glutamine, 100 µM norepinephrine, 100 U/ml penicillin and 100 µg/ml streptomycin in 10 cm plates pre-coated with fibronectin (BD Biosciences, PA). The medium was changed every 24–48 h. Mouse ventricular myocytes isolated from 10 week old FVB mice as previously described [35] were directly subjected to a chromatin immunoprecipitation (ChIP) assay.

### Bioinformatics

The *SCN5a* genomic promoter sequences from mouse, rat, and human were downloaded from the University of California Santa Cruz genome browser [36] and comparative genomic analysis was done with the visualization tools for alignments (VISTA) algorithm (available from the VISTA web site). The Foxo protein biding sequences were compared.

### Transfection and Luciferase Assays

Lipofectamine ™ and plus ™ reagents (Invitrogen) were used to transfect *SNC5a* reporters (*SCN5a* promoter-Luc plasmid [37] was generously provided by Dr. Hideko Kasahara in University of Florida College of Medicine, Florida, USA) and *Foxo1* in the HeLa cells. Cells were seeded in 24-well plates and grown to 90% confluence. Transfections were performed according to the manufacturer's instructions. A *Renilla* reporter gene (Promega) was included as an internal control. Cell lysates were prepared for the luciferase assay 24 hours after transfection as described by the manufacturer (Promega). Data were analyzed and expressed as the normalized-fold changes over controls.

### RT-PCR

Total RNA was extracted from HL-1 and HeLa cells by RNeasy Mini kit (QIAGEN) and reversely transcribed to cDNA with a high capacity cDNA reverse transcription kit (AB Applied Biosystems). Semi-quantitative PCR was conducted to measure mRNA expression levels across different experimental conditions as indicated under “Results”. The primers used to amplify mouse(m) Na_V_1.5, mß-actin, human(h) GAPDH, mGAPDH, hFoxo1, and mTR4 were summarized in table 1.

**Table 1:** Summary of primers for RT-PCR.

	Forward Primer	Reverse Primer
m1Na_V_1.5	5′-ACAGGCCTCCAAAAAGCTGCCAGA-3′	5′-GGGTCGTGTTGTGCCATGAACACA-3′
m2Na_V_1.5	5′-CAGGTCGGAAACTTGGTCTTCAC-3′	5′-GAAGATGATGAAAGTCTCGAACCA-3′
mβ-actin	5′-AGATGTGGATCAGCAG-3′	5′-GCGCAAGTTAGGTTTTGTCA-3′
hGAPDH	5′-ACGGATTTGGTCGTATTGGG-3′	5′-CGCTCCTGGAAGATGGTGAT-3′
mGAPDH	5′-TGAACGGATTTGGCCGTATTGGGC-3′	5′-TCTTCTGGGTGGCAGTGATGGCAT-3′
hFoxo1	5′-AACCTGTCCTACGCCGACCTCA-3′	5′-GCTCGGCTTCGGCTCTTAGCAA-3′
mTR4	5′-GTAGCCTCACCTCAGCGCATTCA-3′	5′-GCGGTTACGGTGGTGCTTGTTG-3′

Note: m, mouse; h, human.

### Western Blotting

HL-1 cells were rinsed with phosphate-buffered saline (PBS) twice, and protein was extracted in cold cell lysis buffer (Cell Signaling). Protein concentration was determined by a detergent-compatible protein assay (Bio-Rad). Equal amounts of protein were resolved by SDS-PAGE, transferred onto nitrocellulose membranes, blocked with 5% non-fat milk for 1 hour, and then incubated with the indicated primary antibody overnight at 4°C. After 1 hour incubation with the appropriate secondary antibody, specific signals were revealed by enhanced chemiluminescence reagent (Pierce). The primary antibodies used were as follows: rabbit anti-Foxo1 polyclonal (Cell Signaling, 1∶1000), rabbit anti-Na_V_1.5 polyclonal (Alomone Labs, 1∶500), rabbit anti-GAPDH polyclonal (Millipore, 1∶4000), and mouse anti-tubulin monoclonal (Sigma, 1∶5000).

### Fluorescen and Confocal Microscopy

HL-1 cells were dispersed on the glass in the center of the 32 mm dishes and grown to 80% confluence. HL-1 cells expressing GFP and Foxo1-GFP were fixed with 4% paraformaldehyde. Nuclear profiles of were revealed with a brief incubation in DAPI (Molecular Probes) prior to microscopic observation. Fluorescence was visualized with an inverted Olympus IX81 fluorescence confocal microscope and photographed for direct import into Image J. All images were processed in an identical manner to faithfully capture the real time images of each sample.

### Adenovirus Vectors

The adenoviral vectors used were as follows: Adv–CMV-FoxO1-AA-GFP expressing constitutively nuclear FoxO1-AA-GFP and Adv-CMV-GFP expressing GFP. Adv–FoxO1-RNAi vector was constructed based on the Block-iT Adenoviral RNAi Expression System (Invitrogen). This FoxO1-RNAi vector encodes a 19-bp DNA (5′-CGCCCCAGGTGGTGGAGAC-3′) that is complementary to the Foxo1 mRNA sequence (10–29 nt) under the control of the mouse U6 promoter. Likewise, a control adenovirus containing the scrambled RNAi (5′-GGACTCGGGCCACCGGGTA-3′) (Adv-scramble-RNAi) under the control of mouse U6 promoter was constructed.

### ChIP Assay

ChIP was used to study the interaction between Foxo1 and *SCN5a* promoter DNA in HL-1 cells, as previously described [38]. Approximately 1×10^7^ HL-1 cells were subjected to the ChIP assay using anti-Foxo1 antibody or rabbit control IgG as a control and the ChIP assay kit (Upstate Biotechnology), as previous described [38]. The immunoprecipitates were analyzed by immunoblot analysis using goat anti-Foxo1 (FKHR-C20; Santa Cruz Biotechnology Inc.), and by PCR assay to detect coimmunoprecipitated DNA using two pairs of *SCN5a* promoter–specific primers (forward 5′-TGGTACATACCGTTTCAGGAC-3′ and reverse 5′ GCACACACACTCACACATAC -3′; forward 5′- TGGTACATACCGTTTCAGGAC-3′ and reverse 5′-ACGCATGCTCACGCACACA -3′) that flank the consensus Foxo1 binding sites (−1484 to −1473 nt) in the mouse *SCN5a* promoter and a pair of primers (forward 5′- GCCTCATTCGTTGCAGTGTGCC-3′ and reverse 5′-GCCAGTGCCTTGTGTGGACTCT-3′) that flanked the DNA sequence (−4453 to −4686) lacking Foxo1 biding site as a control.

### Whole-cell Voltage Clamp Recording

Na^+^ currents were recorded from HL-cells using whole-cell patch-clamp techniques at room temperature (22–24°C). The bath solution for recording membrane currents contained (in mM) 145 NaCl, 4.5 CsCl, 1.5 MgCl_2_, 1 CaCl_2_, 5 HEPES, 5 glucose, 0.1 CdCl_2_ (pH 7.35 with CsOH); the pipette solution contained (in mM) 10 NaF, 110 CsF, 20 CsCl, 10 EGTA, and 10 HEPES (pH 7.35 with CsOH). Electrode resistance ranged from 2.0 to 3.0 MΩ. Data acquisition was carried out using an Axopatch 200B amplifier and pClampex 9.6 software (Axon Instruments Inc., Foster, CA). Currents were acquired at 20–50 kHz (Digidata 1200 A/D converter; Axon Instruments Inc.) and low-pass-filtered at 5 kHz, and stored on a computer. In all recordings, 80% of the series resistance was compensated. Data analysis was accomplished by using pClampfit 9.6 software (Axon Instruments Inc., Foster City, CA).

### Statistical Analysis

All values are expressed as Mean±SEM. Data from experimental groups were compared by student's t-test or ANOVA one way analysis. A value of p<0.05 was considered statistically significant.

## Results

### The Proximal Region of *SCN5a* Promoter Contains Foxo1 Responsive Elements

**Figure 1:**
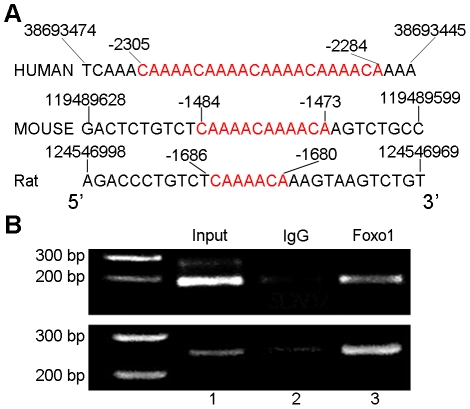
Foxo1 directly binds the insulin response elements in *SCN5a* promoter region. A. Analysis of proximal region of the *SCN5a* promoter showed two overlapping conservative Foxo binding insulin response elements, 5′-CAAAACA-3′, in rat, mouse and human. B. ChIP assay showed that Foxo1 bound *SCN5a* promoter region in both HL-1 cells (upper panel) and adult mouse ventricular myocytes (lower panel) using two sets of paired primers, respectively. Lane 1 and 2 were input and IgG, respectively.

Silico promoter analysis on a list of 354 of the most differentially expressed genes in the thymocyte lineage has revealed putative direct targets that are transcriptionally regulated by Foxo proteins in thymocytes, and that *SCN5a* transcription is upregulated by Foxos deletion [33]. These findings suggest that transcription of *SCN5A* may be directly regulated by Foxos in cardiomyocytes. Analysis of the *SCN5a* promoter region showed a highly conserved DNA sequence (5′-CAAAACA-3′) as IRE in humans and rodents such as mouse and rat. The mouse and human *SCN5a* promoter regions harbor two overlapping Foxo1 binding IREs, 5′-CAAAACAAAACA-3′ (Fig. 1A).

### Foxo1 directly binds *SCN5a* promoter

To confirm the association of Foxo1 with the *SCN5a* promoter region including IREs, a ChIP assay was carried out in HL-1 cells and mouse ventricular myocytes, and it unveiled a clear enrichment of DNA amplified by PCR with two sets of primer pairs flanking Foxo1 binding IREs (5′-CAAAACAAAACA-3′) in the proximal *SCN5a* promoter region following immunoprecipitation with Foxo1 antibody, but not with IgG control (Fig. 1B). Another pair of primers was designed to amplify a region away from Foxo1 binding site, 5′-CAAAACA-3′. As showed in Figure S1, there was no PCR product detected after chromatin immunoprecipitation by Foxo1 antibody.

### Foxo1 Negatively Regulates *SCN5a* Promoter Activity

**Figure 2:**
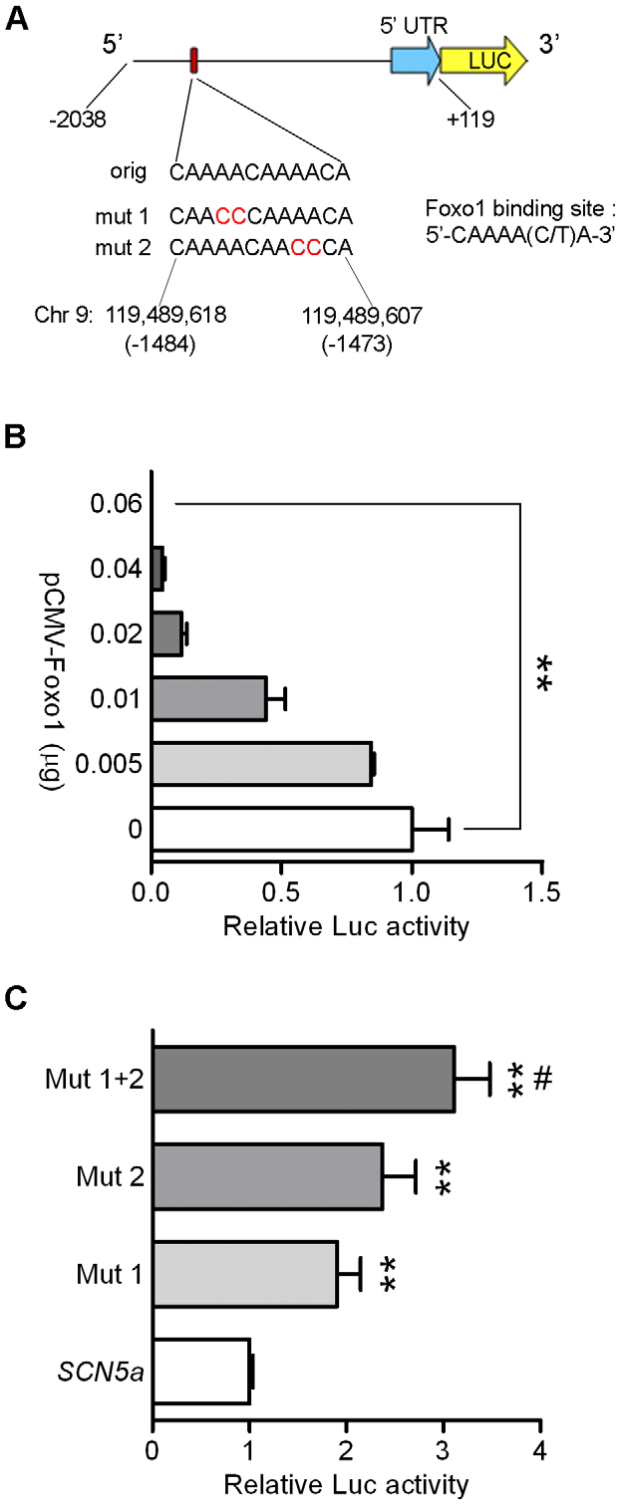
Foxo1 decreases *SCN5a* promoter activity. A. Luciferase constructs contain proximal region of *SCN5a* promoter which has Foxo1 binding insulin response elements and mutations. B. Foxo1 significantly suppressed *SCN5a* promoter activity with dose dependence (**p<0.01, n = 3). C. Individual or double mutations of Foxo1 binding sites in *SCN5a* promoter region prevented Foxo1 inhibition (**p<0.01) compared with wild type *SCN5a*; Double mutation of Foxo1 binding sites had significantly more effective (^#^p<0.05) than mutation 1 (n = 6 in all individual groups).

This conserved sequence is located from −1484 to −1473 in the mouse *SCN5a* promoter region. The proximal promoter region (−2308 to +119 bp) was examined in luciferase reporter assays in HeLa cells with and without expression of Foxo1 (Fig. 2A). Expression of Foxo1 significantly suppressed *SCN5a* promoter activity (p<0.01), and the suppression was Foxo1 dose dependent (Fig. 2B). Individual (CAAAAC to CCCAAC) or double mutations (CAAAACAAAACA to CCCAACCCAACA) in Foxo1 binding sites significantly suppressed (p<0.01) Foxo1 responsiveness compared with wild type *SCN5a*, and double mutations of Foxo1 were significantly more effective (p<0.05) than mutation 1 on *SCN5a* promoter activity (Fig. 2C).

### Foxo1 Negatively Regulates Na_V_1.5 Expression in HL-cells

**Figure 3:**
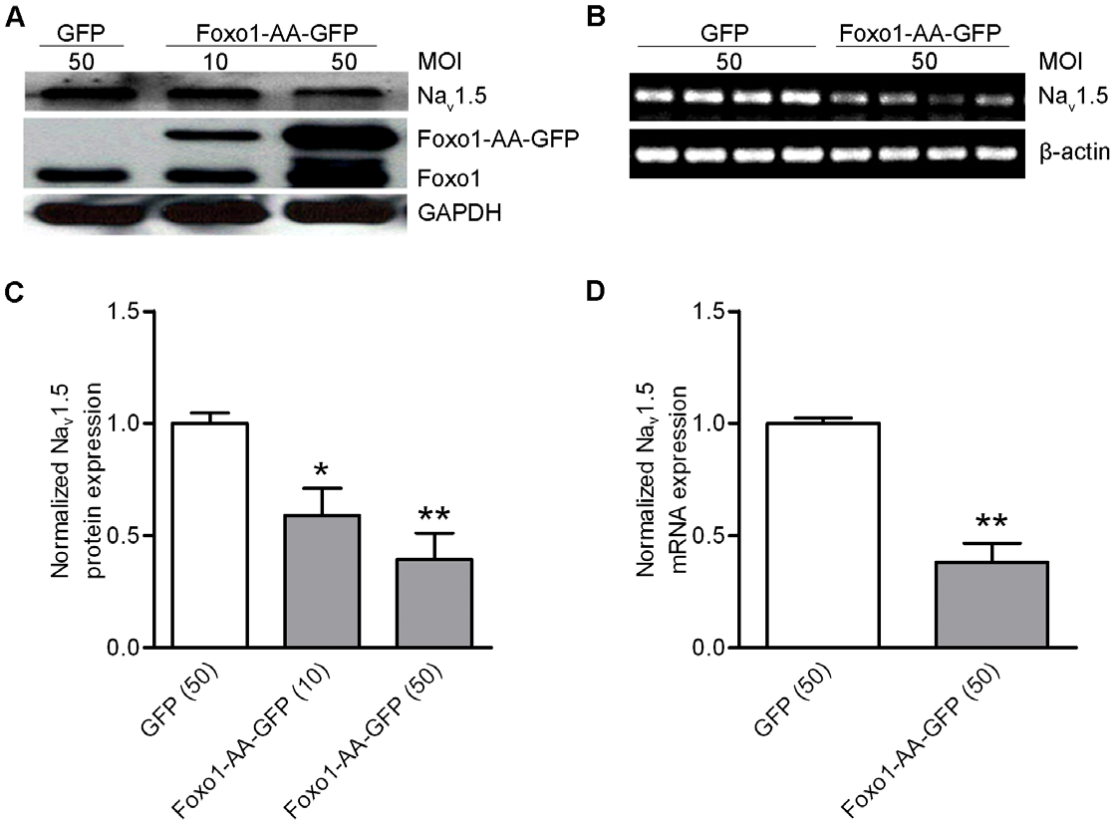
Constitutively nuclear Foxo1 reduced Na_V_1.5 expression. Western blots showed that overexpression of Foxo1-AA-GFP for 36 hours reduced Na_V_1.5 protein level while it increased endogenous Foxo1 expression (A); quantitiation of Na_V_1.5 protein level relative to GAPDH revealed that expression of Foxo1-AA-GFP (n = 5) significantly decreased (*p<0.05, at 10 MOI; **p<0.01 at 50 MOI) Na_V_1.5 expression compared with 50 MOI GFP (n = 5) (C). RT-PCR showed that overexpression of 50 MOI Foxo1-AA-GFP for 36 hours decreased Na_V_1.5 mRNA level compared with that in cells expressing 50 MOI GFP (B) and the values of Na_V_1.5 mRNA relative to ß-actin between GFP (n = 4) and Foxo1-AA-GFP (n = 4) groups were significantly different **(p<0.01) (D).

HL-1 cells harbor robust, rapidly activated and inactivated Na^+^ currents [39], indicating that Na_V_1.5 is expressed in HL-1 cells. In order to test the hypothesis that Foxo1 regulates Na_V_1.5 expression, HL-1 cells were used in the study. The HL-1 cells were infected for 48 hours by 10 or 50 multiplicity of infection (MOI) adenoviruses (Adv)-CMV-Foxo1-AA-GFP [40] and 50 MOI Adv-CMV-GFP. Western blot analysis of the protein extracts from these infected HL-1 cells was performed, and showed that Na_V_1.5 protein was more significantly decreased (p<0.05 or p<0.01) in cells expressing constitutively nuclear Foxo-1-AA-GFP (AA: T24A/S319A) compared to cells expressing GFP (Fig. 3A and C). Interestingly, endogenous Foxo1 was significantly increased by overexpression of Foxo1-AA-GFP (Fig. 3A). This finding is consistent with a previous report that Foxo1 can be positively regulated by Foxo1 binding its own promoter [41]. Accordingly, Na_V_1.5 mRNA level determined by semi-quantitative RT-PCR analysis was also significantly decreased (p<0.01) in HL-1 cells expressing Foxo1-AA-GFP compared to HL-cells expressing GFP (Fig. 3B and D). This pair of primers (m1Na_V_1.5) for RT-PCR application was designed to detect two splicing variants, Na_V_1.5a and Na_V_1.5b [42]. In order to determine if Foxo1 affects the expression of these two splicing variants, the second pair of primers (m2Na_V_1.5) was designed to allow us to detect Na_V_1.5, Na_V_1.5a and Na_V_1.5b. Unfortunately, there was only one band of PCR product at 1300 bp detected, which consistently showed the inhibition of Na_V_1.5 expression by Foxo1-AA-GFP in HL-1 cells (Figure S2). This has prevented us from addressing if Foxo1 also affects the expression of other splicing variant(s).

**Figure 4:**
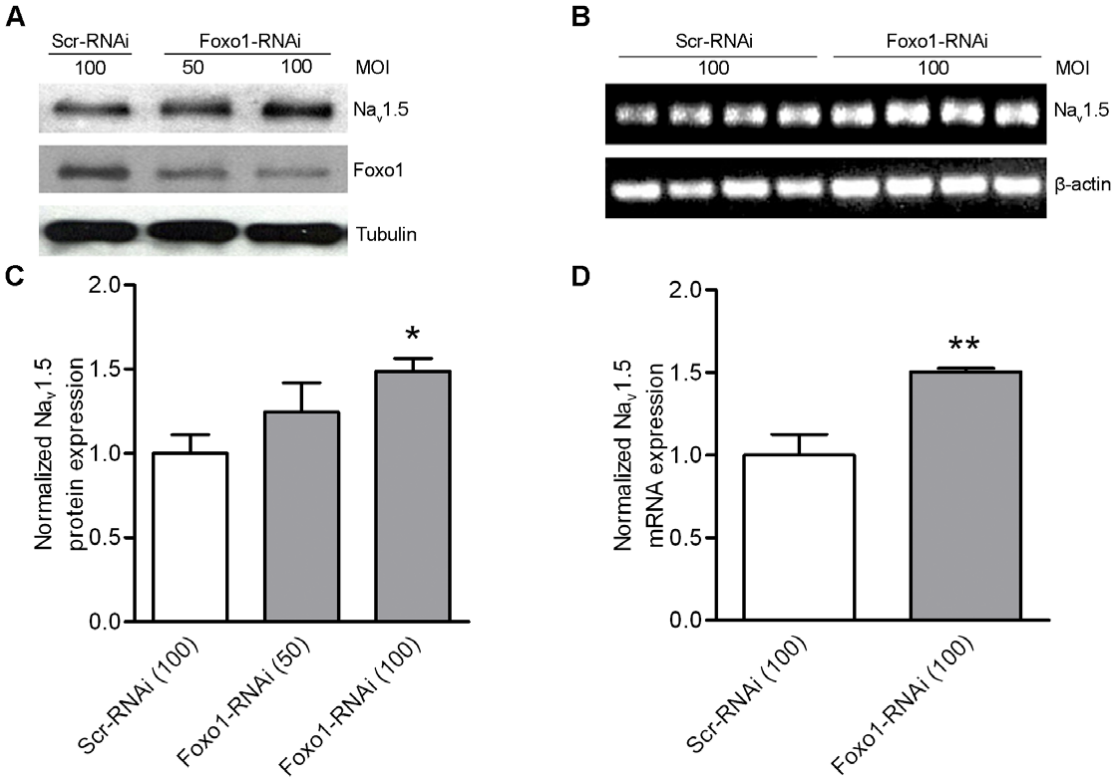
Silencing expression of Foxo1 increases Na_V_1.5 expression. Western blots showed decrease of Foxo1 protein by 50 MOI (n = 4) and 100 MOI Adv-Foxo1 RNAi (n = 4) increased Na_V_1.5 protein in HL-1 cells in comparison with 100 MOI Adv-scramble RNAi (n = 4) (A) and this inhibition reached the significant difference (*p<0.05) at 100 MOI Adv-Foxo1-RNAi (C). RT-PCR showed that Na_V_1.5 mRNA was increased by 50 MOI Foxo1-RNAi (n = 4) in comparison with that in cells expression scramble RNAi (n = 4) (B). The values of Na_V_1.5 mRNA relative ß-actin between Foxo1-RANi and scramble RNAi groups were significant different (**p<0.05) (D).

As shown above, robust Na_V_1.5 expression was observed in the HL-1 cells (Fig. 3A and B). To test the hypothesis that a decrease of endogenous Foxo1 expression upregulates Na_V_1.5 expression, Adv–FoxO1-RNAi was used to infect HL-1 cells to express RNAi and silence Foxo1 expression (Fig. 4A). The downregulation of Foxo1 induced a significant increase in both Na_V_1.5 protein (p<0.05, at 100 MOI) (4A and C) and mRNA levels (p<0.01, at 100 MOI) in HL-1 cells (4B and D) when compared with those obtained from the cells infected with Adv-scramble-RNAi. This FoxO1-RNAi construct encodes a 19-bp oligonucleotide (5′-CGCCCCAGGTGGTGGAGAC-3′) that is complementary to the mouse Foxo1 mRNA sequence (10–29 nt). This sequence has two nucleotides different from that of human Foxo1 mRNA and one nucleotide at the most 5′ different from that of TR4 mRNA -encoded by *Nrc2c* gene. In order to determine the specificity of this RNAi, human HeLa cells expressing endogenous Foxo1 [43] and HL-1 cells were infected with Adv-Foxo-RNAi and Adv-scramble-RNAi, respectively. RT-PCR analysis showed that Foxo1 mRNA in HeLa cells and TR4 mRNA in HL-1 cells were not altered by expression of Adv-Foxo1-RNAi (Figure S3 and S4), indicating that RNAi specifically targeted mouse Foxo1 mRNA.

### Foxo1 Nuclear Localization Reduces Na^+^ Currents in HL-1 cells

**Figure 5:**
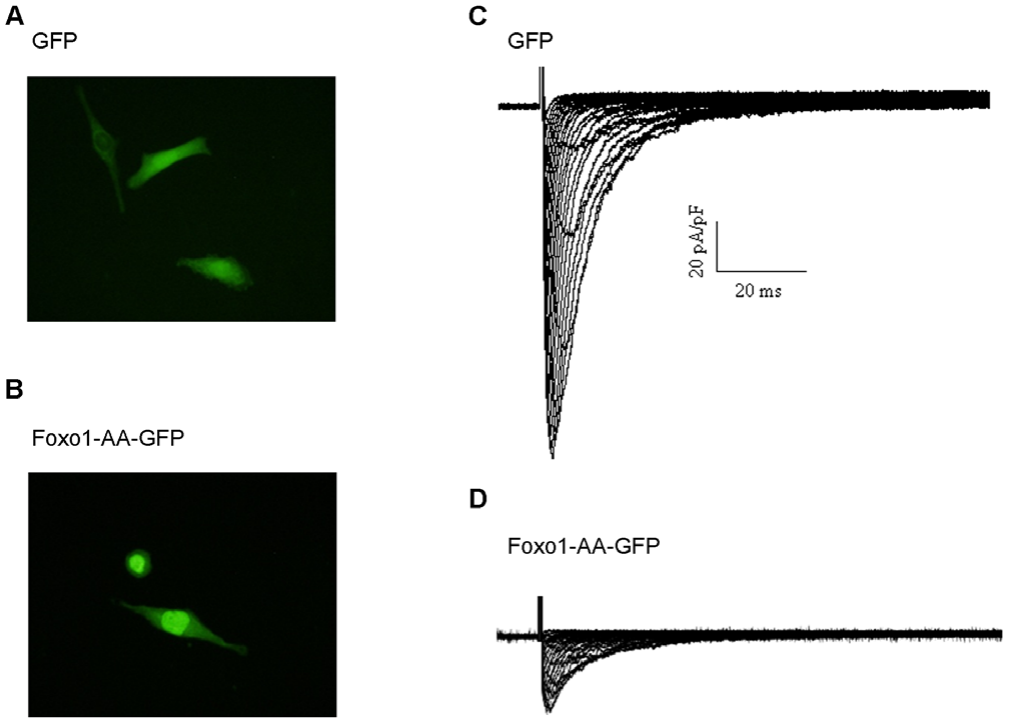
Foxo1 inhibits Na^+^ channel activity. Foxo1-AA-GFP was localized in HL-1 cardiomyocyte nuclei (B) while GFP was seen in both nuclei and cytoplasm (A). Whole cell Na^+^ currents were recorded during 80 ms depolarizing voltage steps to potentials between −60 and +60 mV from a holding potential of −100 mV in HL-1 cells expressing GFP or Foxo1-AA-GFP for 36 hours. The typical whole cell recording traces showed that robust Na^+^ currents were present in HL-1 cells expressing GFP (C) while remarkable reduction of Na^+^ currents in these cells expressing Foxo1-AA-GFP (D).

To test the hypothesis that activation of Foxo1 reducing Na_V_1.5 expression decreases functional Na^+^ currents, whole-cell voltage-clamp Na^+^ current recordings were performed on HL-1 cells infected with 50 MOI Adv-CMV-GFP and 50 MOI Adv-CMV-Foxo1-AA-GFP for 48 hours, respectively. Foxo1-AA-GFP was localized in the nuclei (Fig. 5B), while GFP was seen in both cytoplasm and nuclei (Fig. 5A). Recording traces of rapidly activating and inactivating Na^+^ currents from GFP and Foxo1-AA-GFP-expressing cells, respectively, 36 hours after adenovirus infection, are illustrated in Figure 5C and D. Na^+^ currents were significantly inhibited by expression of Foxo1-AA-GFP, and peak Na^+^ current density was significantly lower (*p*<0.01) in cells expressing Foxo1-AA-GFP (19.6±5.6 pA/pF, at −25 mV) than in cells expressing GFP (67.9±9.6 pA/pF, at −25 mV).

### H_2_O_2_ Promoting Foxo1 Nuclear Localization Reduces Na_V_1.5 Expression

**Figure 6:**
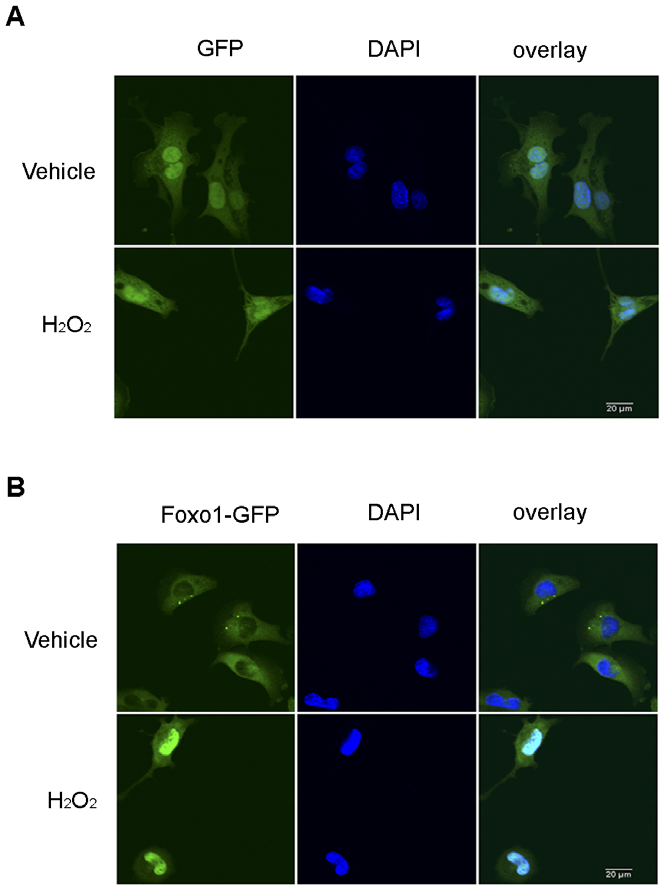
H_2_O_2_ promotes Foxo1 nuclear localization. HL-1 cells were treated with 50 MOI Adv-CMV-GFP or 50 MOI Adv-CMV-Foxo1-GFP for 36 hours and then, 50 µM H_2_O_2_ was added in the medium. GFP was expressed in both cytoplasm and nuclei and its localization was not altered by 2 hour H_2_O_2_ treatment (A) while Foxo1-GFP was expressed in the cytoplasm and 2 hour H_2_O_2_ treatment promoted Foxo1-GFP nuclear localization (B). All images were taken with Ix81 fluorescence confocal microscopy.

*H_2_O_2_ Promotes Foxo1 nuclear localization*: HL-1 cells were infected with 50 MOI Adv-CMV-Foxo1-GFP and 50 MOI Adv-CMV-GFP. These infected cells were cultured for 36 hours. Fifty µM H_2_O_2_ was then added to the culture medium. Fluorescent and confocal images showed that 2 hours of treatment with H_2_O_2_ promoted Foxo1-GFP to localize in nuclei, but not GFP (Fig. 6).

**Figure 7:**
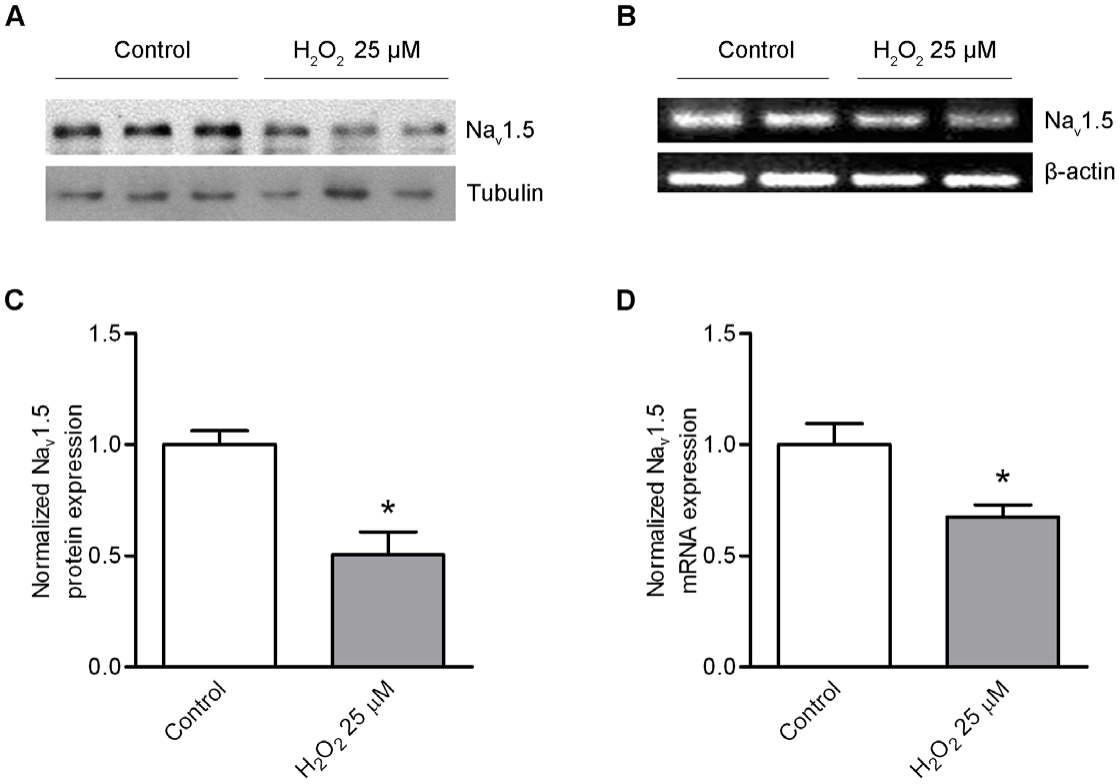
H_2_O_2_ reduces Na_V_1.5 expression. Western blot analysis showed that Na_V_1.5 protein was significantly decreased (*p<0.05) in HL-1 cells treated with 25 µM H_2_O_2_ for 48 h hours (n = 3) compared with the control group (n = 3) (A, C). RT-PCR analysis showed that Na_V_1.5 mRNA significantly reduced (p*<0.05) in HL-1 cells treated with 25 µM H_2_O_2_ for 48 hours (n = 3) compared with the control group (n = 4) (B, D).

**Figure 8:**
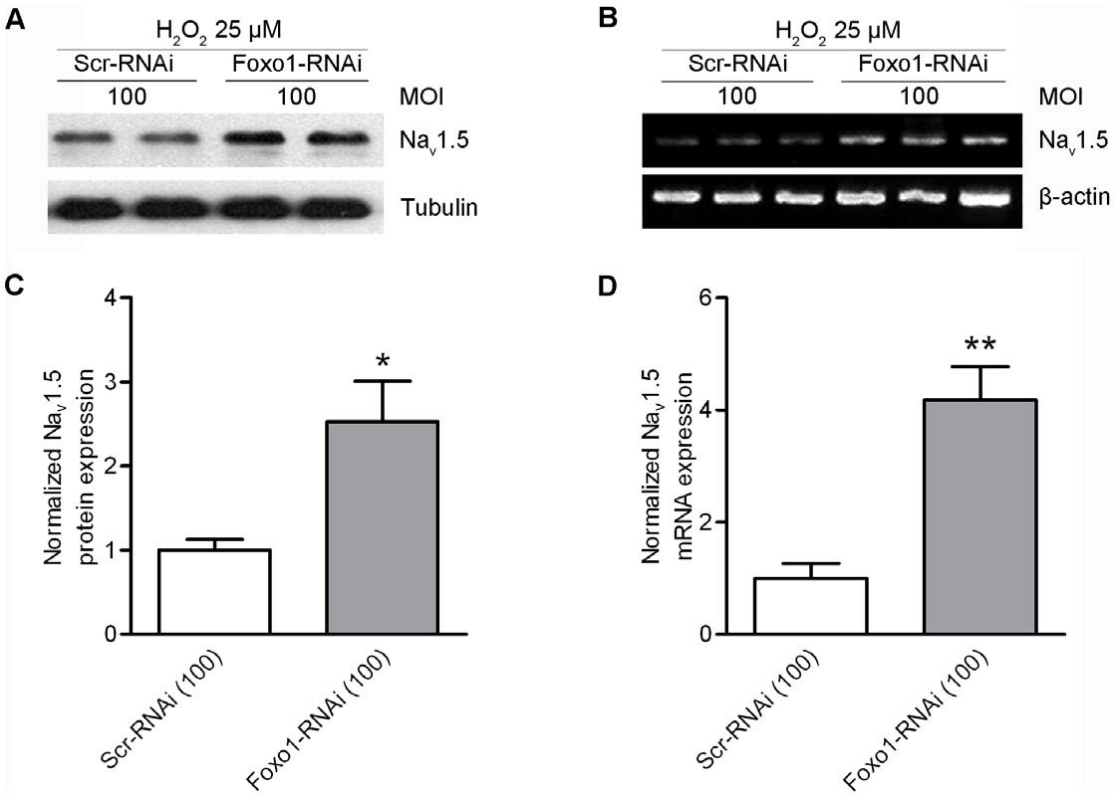
H_2_O_2_ -mediated downregulation of Na_V_1.5 requires Foxo1. Western blot analysis showed that Na_V_1.5 protein was significantly increased (*p<0.05) in HL-1 cells cultured in the medium with 25 µM H_2_O_2_ treatment for 48 hours after the cells expressed Foxo1-RNAi for 24 hours (n = 4) compared with scramble RNAi group (n = 4) (A, C). RT-PCR analysis showed that Na_V_1.5 significantly increased (**p<0.01) in HL-1 cells in the medium with 25 µM H_2_O_2_ treatment for 48 hours after the cells expressed Foxo1-RNAi for 24 hours (n = 4) compared with scramble RNAi group (n = 4) (B, D).

*H_2_O_2_ inhibiting Na_V_1.5 expression is through the Foxo1 signaling pathway*: Increase of Foxo1 nuclear localization by H_2_O_2_ is expected to reduce Na_V_1.5 expression. In order to confirm this assumption, RNA and protein were extracted from HL-1 cells treated with 25 µM H_2_O_2_ for 48 hours. Analyses of RT-PCR products and Western blot results showed that both Na_V_1.5 protein and mRNA levels were significantly decreased (p<0.05) (Fig. 7A, C and B, D) in comparison with controls. In order to determine if H_2_O_2_ reducing Na_V_1.5 expression was through the Foxo1 signaling, 100 MOI Adv-scramble RNAi and 100 MOI Adv-Foxo1-RNAi were used to infect HL-1 cells and 25 µM H_2_O_2_ was added in the culture medium after the cells expressed scramble RNAi and Foxo1-RNAi for 24 hours. Both Na_V_1.5 mRNA and protein levels were significantly increased (p<0.05 and p<0.01, respectively) in the cells expressing Foxo1 RNAi with a 48-hour H_2_O_2_ treatment when compared with the cells expressing scramble RNAi with 48 hour H_2_O_2_ treatment (Fig. 8A, C and B, D).

## Discussion

### Foxo1 negatively regulates Na_V_1.5 expression

Foxo1 is known to function primarily as a transcription factor. The results presented here demonstrate that Foxo1 directly binds the IRE in the *SCN5a* promoter region and inhibits its promoter activity. This inhibition leads to downregulation of Na_V_1.5 expression and a decrease of cardiac Na^+^ channel activity. In the nucleus, Foxo proteins bind a conserved DNA binding sequence, 5′-GTAAA(C/T)A-3′ [30] or IRE, 5′-CAAAA(C/T)A-3′ [30]–[32] in the promoter regions of the targeted genes. Foxo recruitment to the promoter regions usually positively regulates expression of the target genes. For example, Foxo1 bound the consensus sequence in the Kir 6.1 gene promoter and increased the expression of KATP subunit Kir6.1 [25]. In the literature, there is one report in which the authors identified Foxo1 as a corepressor for androgen receptor (AR) [44]. Some corepressors possess intrinsic histone deacetylases (HDAC) activity and some repress gene expression through recruitment of HDAC proteins [45]. It has been shown that HDAC3, but not HDAC1 and HDAC2 associate with Foxo1, enhancing Foxo1-induced inhibition of AR function in LNCaP cells [46]. Whether Foxo1 recruits HADC3 to inhibit the *SCN5a* promoter activity remains undetermined.

### H_2_O_2_ suppressing Na_V_1.5 expression requires Foxo1

ROS have been shown to acutely enhance late Na^+^ currents, leading to action potential prolongation and early afterdepolarizations [47]–[49] via activating CamKII [8], [7] and phosphorylating Na_V_1.5. ROS have also been reported to acutely reduce fast transient outward K^+^ current, leading to the prolongation of cardiac repolarization by phosphorylating Kv4.3 [50]. The chronic effects of H_2_O_2_ on Na_V_1.5 expression were also well studied [9]. Na_V_1.5 expression was suppressed by H_2_O_2_ activating NF-KappaB and partially inhibiting *SCN5a* promoter activity [9]. These findings indicate that other mechanisms are also involved in the regulation of Na_V_1.5 expression at the transcriptional level. As reflected in our studies, H_2_O_2_ reduced Na_V_1.5 expression by promoting Foxo1 nuclear localization, and Foxo1 signaling is required for H_2_O_2_ to affect Na_V_1.5 expression. ROS modulate Foxo1 subcellular localization through different pathways. H_2_O_2_ inhibits phosphorylation of Foxo1 at Ser-253 and The-24 by reducing Akt kinase activity in response to insulin [12]. Under oxidative stress, the activated JNK pathway decreases the activity of Akt in HIT cells, leading to the decrease of phosphorylation of Foxo1 and promoting its nuclear localization [51]. Oxidative stress also augments PRMT1-induced Foxo1 methylation, thereby leading to the inhibition of its phosphorylation by Akt and promotion of its nuclear localization [13]. Sirt1 has been reported to be activated by H_2_O_2_ and the subsequently activated Sirt1 deacetylates and entraps Foxo1 in the nucleus, which is also independent of H_2_O_2_-induced dephosphorylation of Foxo1 [12]. It is likely that Sirt1, Akt, JNK and PRMT1 signaling pathways are involved in the regulation of Na_V_1.5 expression under oxidative stress. Foxo4 has been shown to interact with NF-KappaB and subsequently inhibit its transcriptional activity [52]. Whether Foxo1 suppressing Na_V_1.5 expression in cardiac myocytes is also partially through the NF-KappaB signaling pathway remains to be verified.

### Novelty and Potential Significance

ROS including H_2_O_2_ are significantly elevated in ischemic heart disease (IHD), [1]–[4] in which arrhythmias are frequently observed. A significant decrease in Na^+^ channel activity is considered to be one of mechanisms contributing to the development of arrhythmias in IHD [5], [6]. The mechanisms of transcriptional regulation of Na_V_1.5 expression are largely uncertain. In this study, we demonstrated that Foxo1 directly targeted the *SCN5a* promoter and inhibited its transcriptional activity. Furthermore, we confirmed that H_2_O_2_ promoted Foxo1 nuclear translocation. This Foxo1 relocation is required for H_2_O_2_-mediated Na_V_1.5 downregulation. These findings indicate the important role of Foxo1 in cardiac ion channel regulation and in the development of arrhythmias.

## Supporting Information

Figure S1 Foxo1 does not bind the *SCN5a* promoter region lacking the insulin responsive element. A pair of primers was designed to amplify a region far away from Foxo1 binding site, 5′-CAAAACA-3′. There was no PCR product detected after chromatin immunoprecipitation (ChIP) by Foxo1 antibody (Lane 3). Lane 1 and 2 were input DNA and control IgG ChIP, respectively.
(TIF)

Figure S2 One isoform of Na_V_1.5 is detected in HL-1 cells. RT-PCR using a pair of m2Na_V_1.5 primers showed that there was only one DNA band representative of Na_V_1.5 mRNA after 40 cycles. Overexpression of 50 MOI Foxo1-AA-GFP for 36 hours decreased Na_V_1.5 mRNA level compared with that in cells expressing 50 MOI GFP and the amount of sample loading was equal as determined by GAPDH RT-PCR products.
(TIF)

Figure S3 RNAi targeting mouse Foxo1 mRNA does not affect human Foxo1 expression. RT-PCR using a pair of hFoxo1 primers showed that human Foxo1 mRNA expression was not altered in the HeLa cells infected with 50 MOI Adv-Foxo1-RNAi (n = 3) in comparison with the cells infected 50 MOI Adv-scramble-RNAi (n = 3) (A and B).
(TIF)

Figure S4 RNAi targeting mouse Foxo1 mRNA does not affect TR4 mRNA expression. RT-PCR using a pair of TR4 primers showed that TR4 mRNA expression was not altered in the HL-1 cells infected with Adv-Foxo1-RNAi (n = 3) in comparison with the cells infected Adv-scramble-RNAi (n = 3) (A and B).
(TIF)
