# Supplementary figures and images for: Reactive Oxygen Species Suppress Cardiac NaV1.5 Expression through Foxo1
Source: PLoS One. 2012 Feb 29;7(2):e32738. doi: 10.1371/journal.pone.0032738 (PMC3293505; doi:10.1371/journal.pone.0032738)

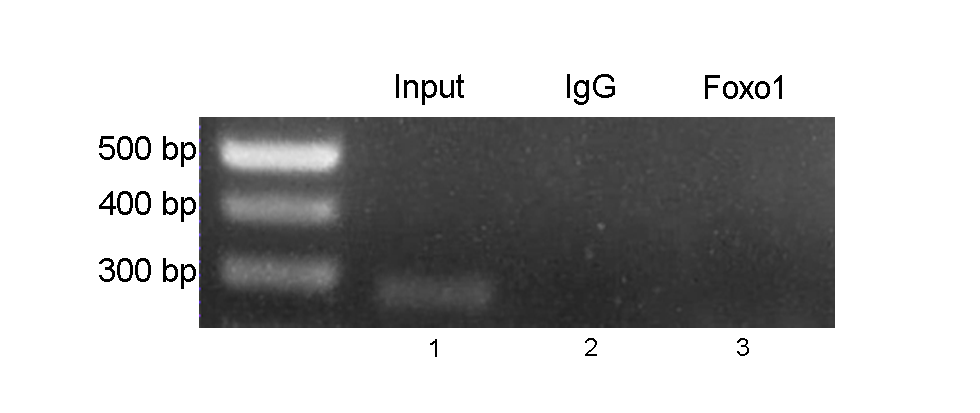

Supplement: Figure S1 — Foxo1 does not bind the SCN5a promoter region lacking the insulin responsive element. A pair of primers was designed to amplify a region far away from Foxo1 binding site, 5′-CAAAACA-3′. There was no PCR product detected after chromatin immunoprecipitation (ChIP) by Foxo1 antibody (Lane 3). Lane 1 and 2 were input DNA and control IgG ChIP, respectively. (TIF) [file pone.0032738.s001.tif]

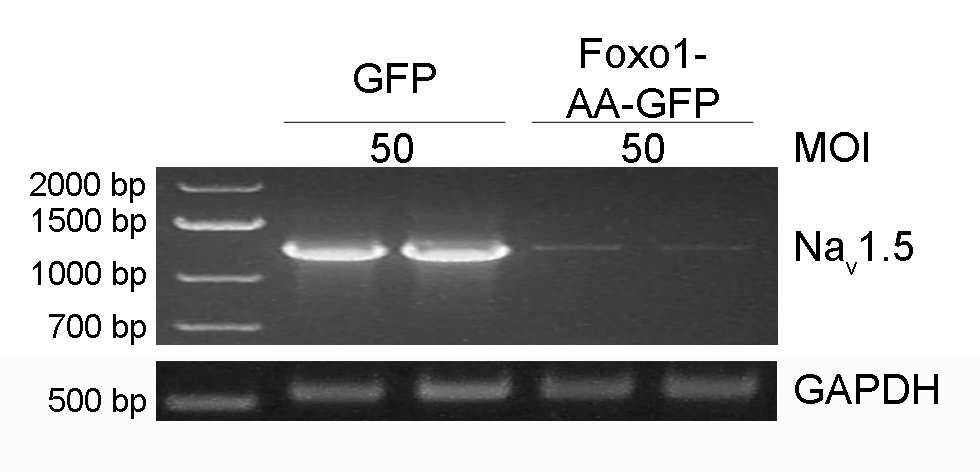

Supplement: Figure S2 — One isoform of NaV1.5 is detected in HL-1 cells. RT-PCR using a pair of m2NaV1.5 primers showed that there was only one DNA band representative of NaV1.5 mRNA after 40 cycles. Overexpression of 50 MOI Foxo1-AA-GFP for 36 hours decreased NaV1.5 mRNA level compared with that in cells expressing 50 MOI GFP and the amount of sample loading was equal as determined by GAPDH RT-PCR products. (TIF) [file pone.0032738.s002.tif]

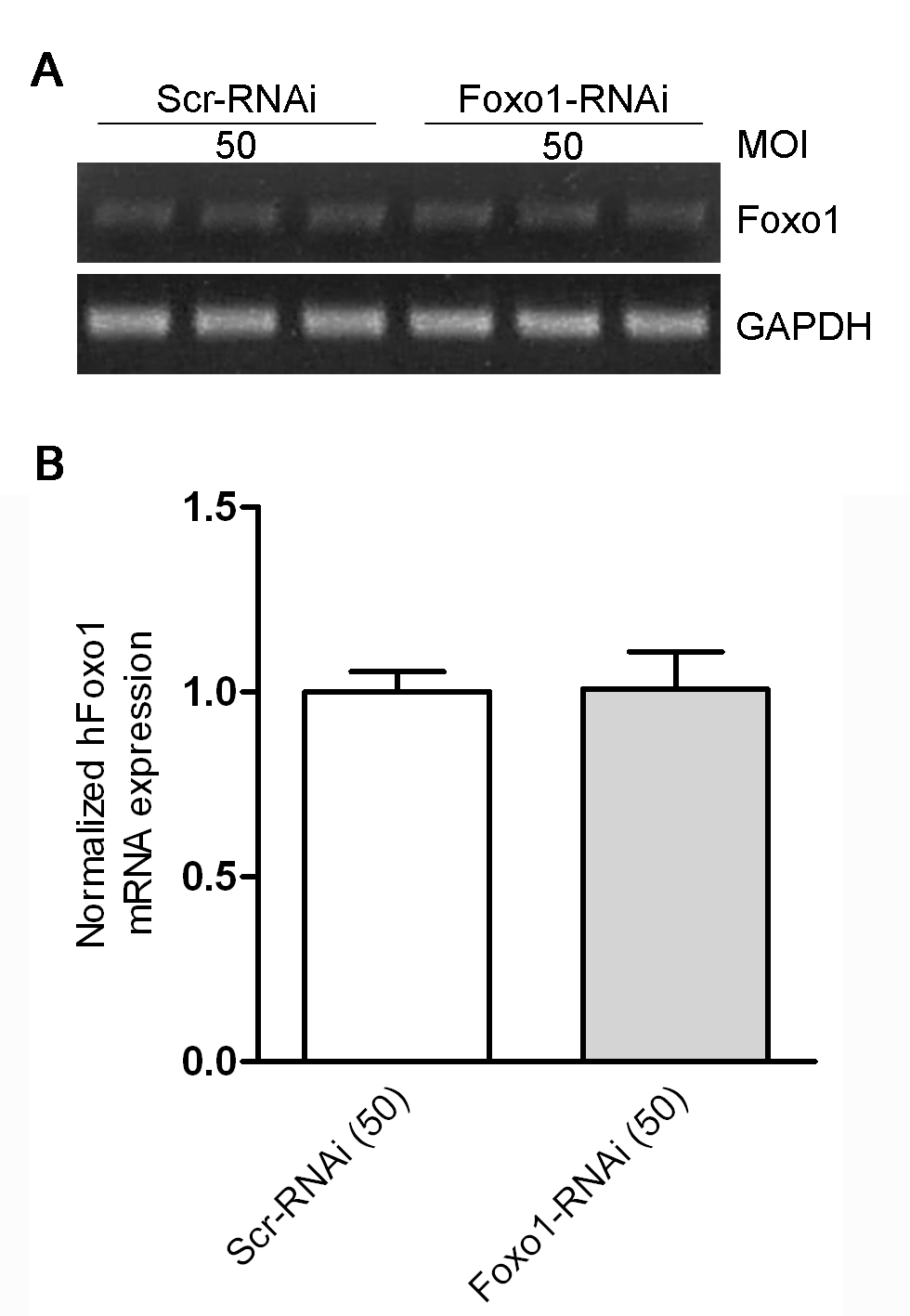

Supplement: Figure S3 — RNAi targeting mouse Foxo1 mRNA does not affect human Foxo1 expression. RT-PCR using a pair of hFoxo1 primers showed that human Foxo1 mRNA expression was not altered in the HeLa cells infected with 50 MOI Adv-Foxo1-RNAi (n = 3) in comparison with the cells infected 50 MOI Adv-scramble-RNAi (n = 3) (A and B). (TIF) [file pone.0032738.s003.tif]

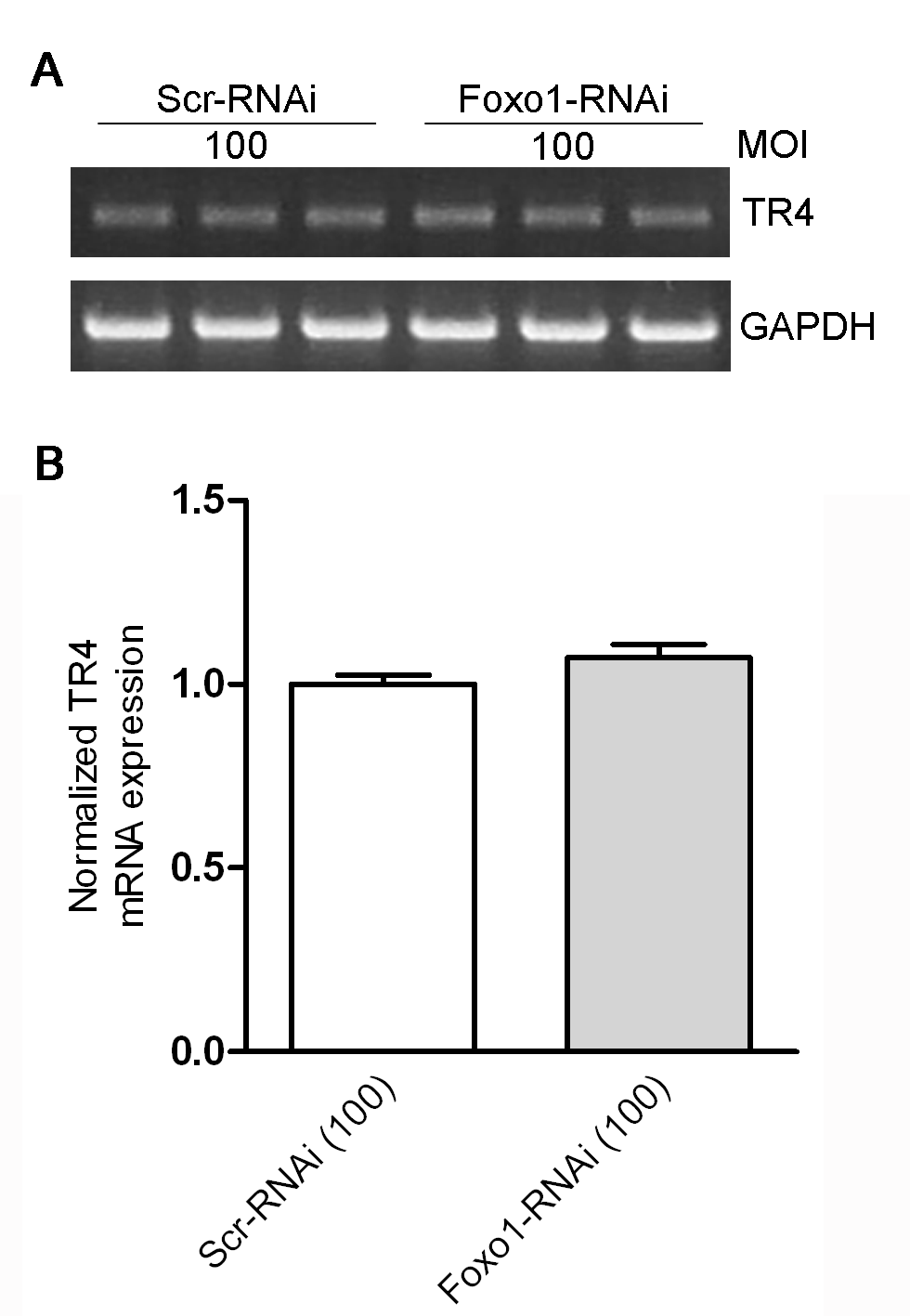

Supplement: Figure S4 — RNAi targeting mouse Foxo1 mRNA does not affect TR4 mRNA expression. RT-PCR using a pair of TR4 primers showed that TR4 mRNA expression was not altered in the HL-1 cells infected with Adv-Foxo1-RNAi (n = 3) in comparison with the cells infected Adv-scramble-RNAi (n = 3) (A and B). (TIF) [file pone.0032738.s004.tif]
